# Ready to leave? – Adolescents’ and parents’ perceptions of transition from paediatric to adult rheumatology care

**DOI:** 10.1186/s12913-024-11265-9

**Published:** 2024-07-10

**Authors:** A. Vermé, Marika Wenemark, J. Granhagen Jungner, E. Broström, C. Bartholdson

**Affiliations:** 1 Department of Women’s and Children’s Health, Karolinska Institutet, Karolinska Vägen 37A, 7 Floor, 171 76 Stockholm, Sweden; 2 Astrid Lindgren Children’s Hospital, Karolinska University Hospital, Stockholm, Sweden; 3 Department of Health, Medicine and Caring Sciences, Faculty of Medicine and Health Sciences, Linköping University, Linköping, Sweden; 4 Unit for Public Health and Statistics, East Region, Linköping, Sweden

**Keywords:** Adolescents, Responsibility, Transition, Transfer

## Abstract

**Background:**

In Sweden, approximately 2000 children live with Juvenile Idiopathic Arthritis (JIA). About half of them continue to have an active disease and need to transfer to adult rheumatology care. This study aimed to investigate Swedish adolescents’ and parents´ perceptions of readiness for transition from pediatric to adult rheumatology care.

**Methods:**

The study was a cross-sectional quantitative study. Patients at the pediatric rheumatology clinic at a university hospital in Sweden and members of The Swedish National Organization for Young Rheumatics aged 14–18 and their parents were invited to participate in the study. Data was collected with the Readiness for Transition Questionnaire (RTQ) focusing on adolescents' transition readiness, adolescents' healthcare behaviors and responsibility, and parental involvement. Data were analyzed with descriptive statistics. Comparative analyses were made using non-parametric tests with significance levels of 0.05 as well as factor analyses and logistic regression.

**Results:**

There were 106 adolescents (85 girls, 20 boys) and 96 parents answering the RTQ. The analysis revealed that many adolescents and parents experienced that the adolescents were ill-prepared to take over responsibility for several healthcare behaviors, such as booking specialty care appointments, calling to renew prescriptions and communicating with medical staff on phone and to transfer to adult care. Parents and adolescents alike stated that it was especially difficult for the adolescents to take responsibility for healthcare behaviors meaning that the adolescents had to have direct interaction with the healthcare professionals (HCPs) at the paediatric rheumatology clinic, for example to renew prescriptions. It was evident that the adolescents who perceived they were ready to take responsibility for the aspects related to direct interaction with HCPs were more overall ready to be transferred to adult care.

**Conclusion:**

Adolescents need more support to feel prepared to transfer to adult care. With the results from this study, we can develop, customize, and optimize transitional care programs in Sweden for adolescents.

## Background

Juvenile Idiopathic Arthritis (JIA) is a heterogeneous group of disorders categorized into seven subgroups, all of which have inflammatory arthritis as a common denominator [1]. JIA can arise at any time during childhood, and girls are more likely than boys to be affected [2]. Yearly, 150–200 children in Sweden are diagnosed with JIA and around 1 500–2 000 children live with the disease [3]. Approximately half of the adolescents with JIA continue to have an active disease as they enter adulthood, and need to transfer to adult care [4, 5]. In Sweden, most adolescents will be transferred to adult care at the age of 18 years. The transfer from paediatric to adult rheumatology care is, for some, perceived as difficult, and can cause anxiety among adolescents and their parents [6].

There is a distinction between transfer and transition. The term ‛transfer’ refers to an occurrence or series of events when adolescents with ongoing physical and medical issues move from receiving care in a paediatric setting to an adult medical setting. ‘Transition’, on the other hand, is a process that occurs throughout adolescence and aims to educate adolescents to manage their lives and health in relation to their chronic illness. The learning process should start before the adolescent enters puberty, and last until they are old enough to take care of themselves [7]. The Society for Adolescent Medicine defines transitional care as “the purposeful, planned movement of adolescents and young adults with chronic physical and medical conditions from child-centred to adult-oriented healthcare systems” (p.570) [8].

Previous research shows that the transition process gives the best results if initiated as soon as the child enters adolescence. It can begin as early as 11 years of age but not later than the age of 14 [9]. The necessity of starting the process in early adolescence was also demonstrated by McDonagh and colleagues, who examined a coordinated evidence-based approach. Twelve months following the program’s implementation, the youngest group (aged 11) displayed significant gains in knowledge of arthritis, an increase in self-medication, and increased satisfaction with rheumatology care [10].

In the transition process, it is important to include the parents and to help them adjust to their new role as they will no longer be the ones who have the main responsibility for the adolescent’s healthcare [11]. During the transition process, healthcare professionals (HCPs) should try to strengthen the adolescent’s independence without undermining the parent’s role [12]. HCPs, adolescents, and parents must try to create a common view on how the transition process facilitates and strengthens the adolescent’s independence [13].

There are several methods for measuring readiness for transition targeting adolescents, parents, and HCPs. In the literature, both quantitative [14–17] and qualitative [18–21] methods are used. In Sweden the Readiness for Transition Questionnaire (RTQ) has recently been used [26]. Results from previous research measuring readiness show that both adolescents and parents need more knowledge about transition. In a study with 49 patients suffering from JIA and 103 parents, Matsumoto (2021) shows that over half of the adolescents and about one-third of their parents had limited knowledge about what transitional care was, and over half of the adolescents and nearly four-fifths of the parents felt worried to transfer to adult care [22]. This study also showed that about half of both the adolescents and the parents were not given the opportunity to talk to the doctor about the upcoming transfer to adult care. A major worry was that the medical doctors in adult care would not have adequate knowledge about JIA [22, 23]. In another study by Sömnez (2021), of 157 patients with different rheumatology diagnoses and their parents, half of the adolescents and almost all the parents wanted to stay in paediatric care [23] and were, accordingly, not sufficiently ready for transfer. Moreover, age seems to be an important determinator for perceptions of readiness for transfer from paediatric rheumatology [24]. Bingham and colleagues found that older children had higher self-reported autonomy in most questions asked regarding accessing medical care [25]. Other patient characteristics associated with high self-perceived autonomy included having a family member with a similar disease, having a younger parent, and longer disease duration [25].

Transfer to adult rheumatology is under-researched [10]. In Sweden, no studies have been made on readiness for transfer to adult care among adolescents with JIA and there is no structured transition program or guidelines for this patient group. In order to tailor and improve transitional care for adolescents with JIA, it is essential to know how ready adolescents and their parents feel about different aspects of the transition and, ultimately, the transfer from paediatric to adult care. Investigation of adolescents’ and parents’ readiness could facilitate understanding of the transition process and create foundations for individualized support.

### Aims

The aim of this study was to investigate Swedish adolescents with JIA and their parents’ perceptions of readiness for transition from paediatric to adult rheumatology care.

## Methods

### Study design

A cross-sectional quantitative study.

### Participants

Data were collected from March 2020 to March 2022. Patients at the paediatric rheumatology clinic at a university hospital in Stockholm and members of the Swedish National Organization for Young Rheumatics were invited, together with their parents, to participate in the study.

### Inclusion criteria

Confirmed Juvenile Idiopathic Arthritis, adolescents turning 14 the year of inclusion and adolescents up to 18 years.

### Exclusion criteria

Uncertain paediatric rheumatic disease. Non-Swedish speaking adolescents/parents.

### Questionnaire

The readiness for transition questionnaire (RTQ) was originally developed and validated by Gilleland [15] for patient groups with different chronic diagnoses, and is also available in a parental version [15]. The questionnaire has been translated and culturally adapted into Swedish [26] using scientific guidelines [27]. The first part of the RTQ covers four questions about responsibility for healthcare [15]. Furthermore, the RTQ covers adolescent responsibility and parental involvement in different healthcare-related behaviours, as well as final questions about overall transition readiness and overall readiness for responsibility for healthcare [15]. Included healthcare-related behaviours are, for example, regular blood samples, taking medications, and being in contact with the clinic. The questions about healthcare-related behaviours have five possible answers for adolescent responsibility: 1 = Not responsible at all; 2 = Sometimes responsible; 3 = Often responsible; 4 = Almost always responsible; and 5 = Not relevant. The same behaviours are also investigated in the section about parental involvement, with response options as follows: 1 = Not involved at all; 2 = Sometimes involved; 3 = Often involved; 4 = Almost always involved; and 5 = Not relevant. The response option *Not relevant* was added for questions related to healthcare behaviours during the adaptation process [26]. Response alternatives to the final overall questions are: Not at all ready; Somewhat ready; Mostly ready; and Completely ready.

In the present study, two minor adjustments were made to the Swedish version [26], in both the adolescent and parent versions: 1) the word “daily” was excluded from the question about daily medications since not all medications are taken daily within paediatric rheumatology, and 2) the example of type of clinics in the question about scheduling specialty care appointments was replaced by clinics more relevant to the patient group. Furthermore, questions (*n* = 5) about health were excluded due to ambiguities in what adolescents include in the health concept, and that the original questionnaire [15] focused on healthcare and not health.

### Data collection

In total, 225 members of the Swedish National Organization for Young Rheumatics and 110 patients from the paediatric rheumatology clinic were invited to participate in the study by a research invitation letter sent to their home addresses. The research invitation letter included information explaining the purpose of the study and separate QR codes for adolescents and parents to access the digital anonymous RTQ. One reminder was sent to the intended study participants at one point.

### Ethical approval declarations

The study was approved by the Ethical Review Board in Sweden Dnr 2019–01540.

### Data analysis

Data were analysed with descriptive statistical methods calculating percentages, medians, ranges, means, and standard deviations. Comparative analyses were made using non-parametric tests (Mann Whitney U test) with a significance level of 0.05. The two parts concerning adolescent responsibility and parental involvement for different healthcare-related behaviours were combined to obtain a measure of the adolescent’s level of “independent responsibility”. In the combined measure, no distinction was made between the two response options “Often” and “Almost always” since we found it difficult to distinguish between them. The independent responsibility measures are described below:

- *No responsibility* – Adolescent not responsible at all.
- *Minor responsibility* – Adolescent sometimes responsible.
- *Shared responsibility* – Adolescent often responsible with parents often involved.
- *Major responsibility* – Adolescent often responsible with parents sometimes or not at all involved.

Factor analysis (Varimax rotated principal component analysis) was used to investigate the dimensions of the items. Logistic regression was used to analyse what factor(s) explain adolescents feeling almost fully or fully ready to transfer to adult care.

## Results

**Fig. 1:**
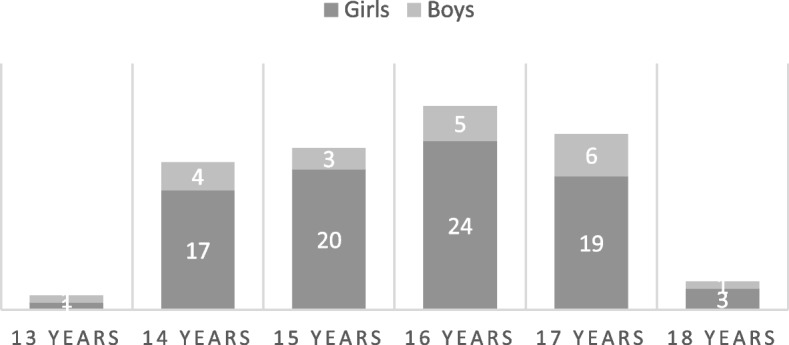
Age distribution in the adolescent group (*n* = 104)

Out of 335 patients receiving the invitation, including an invitation to their parents, 106 adolescents (girls *n* = 85, boys *n* = 20, one missing) and 96 parents answered the questionnaire. The response rate was 32% for adolescents but unknown for parents since information about the number of parents in each family receiving the invitation is missing. Adolescents’ ages ranged from 13 to 18 years old (Fig. 1) with a median age of 16 years (participants who were 13 years old were turning 14 the same year). Sociodemographic questions in RTQ include age and gender but not diagnosis subgroup. Therefore, we do not know which subgroup of JIA the participants had. The clinic treats patients with all types of JIA and therefore it can be assumed, but not certain, that there also were a spread of JIA sub group diagnosis among the respondents.

### Responsibility and involvement in different healthcare-related behaviours

Adolescents and parents were asked about how much responsibility the adolescents took for different healthcare-related behaviours, and how much the parents were involved in, for example, taking medication, renewing prescriptions, and attending medical appointments (Table 1). In general, 16–18-year-old adolescents reported taking more responsibility and having lower parental involvement than 13–15-year-olds. For several behaviours, there were significant differences between the two age groups of adolescents (Table 2). When analysing gender, one significant difference emerged; namely, girls reported more responsibility for communication with the clinic on the phone compared to boys (p value = 0.045) (Table 2). The adolescents reported themselves as taking greater responsibility than the parents reported them as doing. However, both adolescents and parents reported equally that the adolescents took less responsibility for tasks involving direct contact with the clinic (Table 1). Both adolescents and parents reported that the parents were largely involved in healthcare-related behaviours. The younger adolescents (13–15 years) reported that their parents were more involved in healthcare-related behaviours than the older adolescents (16–18 years) reported (Table 1).

**Table 1:** Adolescents’ and parents’ assessment of adolescent responsibility and parental involvement

Healthcare-related behaviours	Adolescence responsibility			Parental involvement		
	Ad 13–15 y Mean (SD)	Ad 16–18 y Mean (SD)	Parents Mean (SD)	Ad 13–15 y Mean (SD)	Ad 16–18 y Mean (SD)	Parents Mean (SD)
Getting regular labs	2.37 (1.17)	2.87 (1.01)	2.52 (1.25)	3.76 (0.59)	3.17 (0.99)	3.60 (0.78)
Taking medications	3.10 (0.85)	3.34 (0.90)	2.92 (1.06)	3.51 (0.68)	3.12 (0.95)	3.46 (0.77)
Booking specialty care appointments	1.47 (0.80)	1.88 (0.99)	1.61 (0.93)	3.86 (0.35)	3.65 (0.72)	3.74 (0.61)
Booking primary care appointments	1.33 (0.72)	1.87 (0.97)	1.45 (0.79)	3.90 (0.31)	3.64 (0.65)	3.76 (0.65)
Calling to renew prescriptions	1.16 (0.50)	1.75 (0.91)	1.37 (0.84)	3.88 (0.40)	3.58 (0.88)	3.67 (0.79)
Explaining disease to others	3.20 (0.85)	3.22 (0.83)	2.70 (1.01)	2.93 (0.81)	2.79 (0.91)	3.33 (0.80)
Attending medical appointments	2.76 (1,10)	3.30 (0.94)	3.05 (1.06)	3.77 (0.52)	3.50 (0.83)	3.66 (0.65)
Communicating with medical staff in person	3.40 (0.71)	3.27 (0.70)	3.13 (0.95)	3.47 (0.72)	3.35 (0.73)	3.48 (0.79)
Communicating with medical staff on phone	1.59 (0.80)	2.18 (1.04)	1.84 (1.13)	3.71 (0.60)	3.33 (0.92)	3.37 (0.85)

**Table 2:** Overview of significant differences

Responsibility for healthcare behaviours	Mean (SD)		*P*-value
*Differences between adolescents’ age groups*	Ad 13–15 y	Ad 16–18 y	
Getting regular labs	2.37 (1.17)	2.87 (1.01)	0.030
Booking specialty care appointments	1.47 (0.72)	1.88 (0.99)	0.028
Booking primary care appointments	1.33 (0.72)	1.87 (0.97)	0.004
Calling to renew prescriptions	1.16 (0.50)	1.75 (0.91)	< 0.001
Attending medical appointments	2.76 (1.10)	3.30 (0.94)	0.019
Communicating with medical staff over the phone	1.59 (0.80)	2.18 (1.04)	0.009
*Differences between gender*	Girls	Boys	
Communicating with medical staff over the phone	2.04 (0.99)	1.56 (0.86)	0.045
*Differences between adolescents and parents*	Adolescents	Parents	
Taking medications	2.64 (1.10)	2.92 (1.06)	0.043
Calling to renew prescriptions	1.51 (0.81)	1.37 (0.84)	0.049
Explaining disease to others	3.22 (0.83)	2.70 (1.06)	< 0.001
Parental involvement in healthcare behaviours			
* Differences between adolescents’ age groups*	Ad 13–15 y	Ad 16–18 y	
Taking regular labs	3.76 (0.59)	3.17 (0.96)	0.001
Taking medications	3.51 (0.68)	3.12 (0.95)	0.032
Booking visits to primary care	3.90 (0.31)	3.64 (0.65)	0.048
Communicating with medical staff over the phone	3.71 (0.60)	3.33 (0.92)	0.040
* Differences between adolescents and parents*	Adolescents	Parents	
Explaining disease to others	2.84 (0.87)	3.33 (0.80)	< 0.001
Overall readiness for transfer to adult care			
* Differences between adolescents’ age groups*	Ad 13–15 y	Ad 16–18 y	
Overall readiness to transfer to adult care	1.63 (0.757)	2.14 (0.913)	0.005

### Adolescents’ and parents' perception of independent responsibility regarding different healthcare-related behaviours

**Fig. 2:**
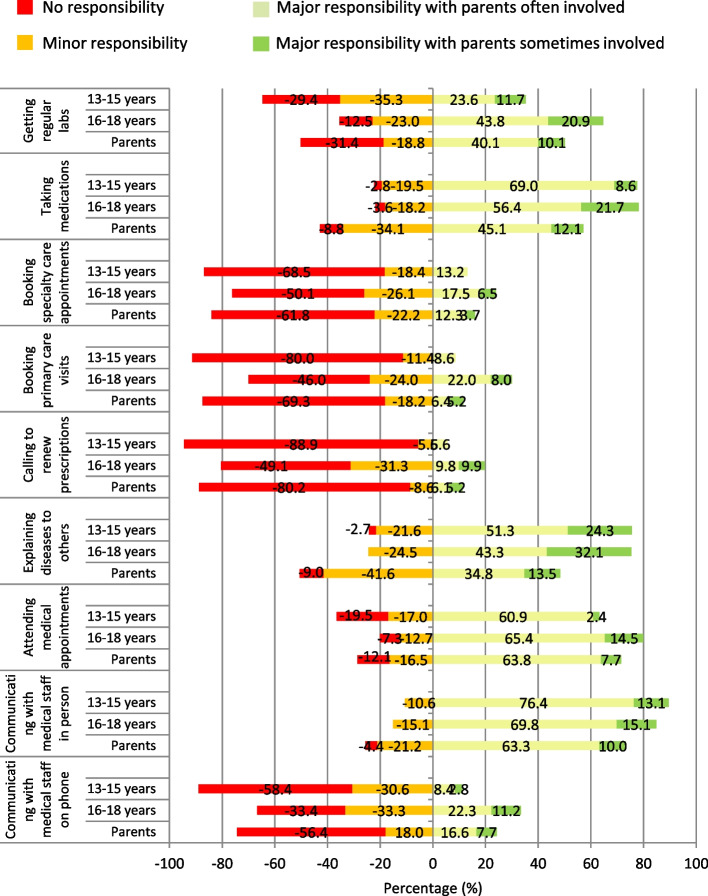
Independent responsibility (a combined measure of adolescent responsibility in relation to parent involvement) for different healthcare-related behaviours

In the combined measure of responsibility with different levels of parental involvement, the analysis showed that direct contact with the healthcare system was most challenging for adolescents (Fig. 2). In many of the other healthcare-related behaviours, for example, taking medication, getting regular labs, and explaining the disease to others, the adolescents perceived themselves as taking major responsibility, while perceiving parents as often involved. A small proportion of adolescents perceived themselves as taking major responsibility without parental involvement. The substantial progress in responsibility, compared between the younger and older adolescent groups, was for getting regular labs and attending medical appointments. For the more challenging activities, there was progress between the younger and older age groups, but still, less than 25% of the older adolescents perceived major responsibility, with parents often/sometimes involved.

### Adolescents’ and parents’ perceptions of overall readiness to transfer to adult care

There was a significant difference (*p* value = 0.005) (Table 2) between the age groups when reporting perceptions of overall readiness to transfer to adult care. In the younger group (13–15 years), 2% perceived that they were fully ready to transfer to adult care compared to 7% of the adolescents in the older group (16–18 years) (Table 3). Moreover, in the younger age group (13–15 years), 48% perceived that they were not ready to transfer to adult care compared to 24% in the older age group (16–18 years). There were no significant gender differences when adolescents reported perceptions on how fully ready they were to transfer to adult care.

**Table 3:** Adolescents’ and parents’ perceptions of the adolescents’ overall readiness to transfer to adult care

	Girls 13–15 years	Boys 13–15 years	All 13–15 years	Girls 16–18 years	Boys 16–18 years	All 16–18 years	All adolescents	All parents
	% (n)	% (n)	% (n)	% (n)	% (n)	% (n)	% (n)	% (n)
Not ready	47 (18)	50 (4)	48 (22)	27 (12)	9 (1)	24 (13)	35 (37)	49 (47)
Rather ready	40 (15)	13 (1)	35 (16)	36 (16)	36 (4)	36 (20)	36 (38)	35 (34)
Almost ready	8 (3)	13 (1)	9 (4)	21 (9)	27 (3)	21 (12)	16 (17)	10 (10)
Fully ready	0 (0)	13 (1)	2 (1)	5 (2)	18 (2)	7 (4)	5 (5)	3 (3)
Missing	5 (2)	13 (1)	7 (3)	11 (5)	9 (1)	11 (6)	9 (9)	2 (2)

Parents’ perceptions are congruent with the younger age group of adolescents. Three percent reported that they perceived their adolescents as being fully ready to transfer and half (49%) of the parental group reported that they did not perceive their adolescents as ready to transfer (Table 3).

### Factors influencing adolescents’ and parents’ perception of overall readiness to transfer to adult care

A factor analysis revealed two factors with eigenvalues above 1, explaining 63.9% of the variance (Table 4). Factor 1 includes variables that have to do with bookings and communication by telephone, conceptualized as “Administration”. Factor 2 includes handling labs/medications and communicating in person, which seem related to engagement and routines of the healthcare, conceptualized as “Engagement”.

**Table 4:** Factor analysis of the nine items of independent responsibility

	Factor 1 Administration	Factor 2 Engagement
Getting regular labs	0.37	0.66
Taking medications	0.15	0.66
Booking specialty care appointments	0.87	0.22
Booking primary care appointments	0.89	0.21
Calling to renew prescriptions	0.86	0.23
Explaining disease to others	0.18	0.68
Attending medical appointments	0.29	0.71
Communicating with medical staff in person	0.10	0.71
Communicating with medical staff on phone	0.78	0.28

The engagement factor gets high scores in the group that feels not ready for transfer and increases for the group that feels rather ready. Thus, it seems that this factor includes the first steps to independent responsibility for healthcare. Adolescents’ and parents’ results are in reasonable agreeance for Factor 2 (Table 5). The administrative factor is continuously increasing in the three levels of readiness, and there is a bigger gap between the group that feels rather ready and the group that feels almost/fully ready. Parents especially don’t seem to judge their adolescents as fully ready for transfer until they also show independent responsibility for administrative matters.

**Table 5:** Factor scores (FS) and confidence interval (CI) for the three different levels of readiness according to adolescents and parents

Overall readiness	Adolescents		Parents	
	Factor 1 Administration (*n* = 81)	Factor 2 Engagement (*n* = 82)	Factor 1 Administration (*n* = 87)	Factor 2 Engagement (*n* = 80)
	FS (CI)	FS (CI)	FS (CI)	FS (CI)
Not ready	1.27 (0.08)	2.62 (0.16)	1.15 (0.05)	2.23 (0.09)
Rather ready	1.72 (0.16)	2.90 (0.08)	1.71 (0.15)	2.82 (0.08)
Almost/Fully ready	2.20 (0.26)	3.12 (0.10)	2.59 (0.25)	3.19 (0.12)

Separate logistic regressions for adolescents and parents showed that only Factor 1 (administrative) was significant for explaining being almost/fully ready for transfer when also controlling for Factor 2 (engagement) (Table 6).

**Table 6:** Logistic regression for being almost/fully ready for transfer to adult care

	Adolescents (*n* = 72)	Parents (*n* = 77)
	Exp B (*p*-value)	Exp B (*p*-value)
Factor 1 Administration	3.09 (*p* = 0.028)	3.49 (*p* = 0.012)
Factor 2 Engagement	4.63 (*p* = 0.13)	6.98 (*p* = 0.084)

## Discussion

This cross-sectional quantitative study’s analysis reveals that many adolescents with JIA were ill-prepared to transfer to adult care. The same issue was reported by their parents. Parents and adolescents alike stated that it was difficult for the adolescents to take responsibility for several healthcare-related behaviours connected to adolescents’ direct interaction with the HCPs at the paediatric rheumatology clinic. It was evident that the adolescents who perceived they were ready to take responsibility for the aspects related to direct interaction with HCPs were more ready to be transferred to adult care.

As mentioned above, challenging healthcare-related behaviours for adolescents included them having direct contact with HCPs, e.g., calling to book an appointment or renew prescriptions. The same results have been shown in other studies [24, 26]. The reasons for these results are not clear. One could argue that the feeling of uncertainty and the fear of making mistakes could be one explanation. Research has shown that adolescents transitioning from adolescent to adult care felt anxious, uncertain, and fearful [28], which could reinforce the fear of making mistakes. The two items that involve using the phone – to communicate with HCPs and renew prescriptions – seem especially difficult for adolescents. There may be several reasons why young people find it challenging to contact healthcare providers by phone. One reason may be that the phone hours are during the day when they are at school, which makes it difficult for them to handle the contact on their own without having to leave classes. Another reason may be that adolescents rarely make telephone calls, and are especially uncomfortable talking to adults on the phone. On the other hand, adolescents are used to using their mobile phones for many other things and it is an environment they feel safe in, which could be argued would perhaps contribute to them feeling comfortable to contact healthcare providers. Healthcare clinics caring for adolescents must fulfill their task to be adolescent-friendly and customize accessibility according to adolescents’ preferences. Using digital platforms, including communication pathways such as chats, could be one solution and would increase flexibility regarding contact times with HCPs. The digital opportunities that exist today could, perhaps, also be utilized in the transition work itself. In a study by Miller [29], it was shown that adolescents who were given the opportunity to use digital transition support by an app on their phone increased their self-confidence in taking care of their illness, and the proportion of those who took responsibility for booking visits to the healthcare system. They also used the app to increase their knowledge of their disease [29].

The present study also provides us with the knowledge that this group of adolescents did not feel ready to transfer to adult care. Only a small percent (5%) of the adolescents reported that they were fully ready to transfer. Our results demonstrate that neither does it seem to be enough to take responsibility for some possibly simpler behaviours to feel fully ready. We speculate that the challenge is, perhaps above all, to start taking responsibility for ‛adult’ things, like booking and calling, i.e., the behaviours included in factor 1. However, it may be difficult for adolescents to evaluate whether they are ready to be transferred, as the idea of it may be abstract and they may not know what to expect. They might not know geographically where they are going, which medical doctor to see, and how the care is conducted there. Despite years of increased study and policy focus on the topic of transition, there are still unmet requirements for adolescents and their families. As a crucial component of an adolescent’s development, health transitions take place concurrently with, and in relation to, a variety of other significant transitions, like transitioning from childhood to being an adolescent, that has an impact on many different facets of life [30], and which may complicate the process further.

Additionally, our analysis reveals that the levels of readiness increase with the age of the adolescents. This result was expected and has been described by others [30], and is most likely associated with the developmental process of going from adolescence to adulthood [31].

In this study, parents graded the adolescents less ready for the transfer than the adolescents graded themselves. Similar results have been reported in other studies investigating transition among adolescent’s with chronic diseases [26]. Only 13% of parents reported that their adolescent was almost or fully ready to transfer, which is low even compared to the youngest age group’s own perception of readiness. The results could be related to a variety factors. One possibility is that adults and adolescents interpret “fully ready” different. Some adolescents may, for example, express readiness without realizing the impact of more independent responsibility that their parents may include in their interpretation of readiness. However, the results may also be an effect of parents underestimating the adolescent’s knowledge and ability. This could mean that adolescents never develop abilities to take responsibility if parents, for example, continue to administer medications and communicate with healthcare providers [32]. It is therefore important for HCPs to enable adolescents to increase their abilities and put them to use to support positive adolescent development. For instance, adolescents could practice asking questions about the care or sensitive topics if they are given the chance to do so while their parents are present. Although the group of boys is small, the results show that boys seem to perceive themselves as ready for transfer to a greater degree than girls. In the present study population, this perhaps can be explained by gender roles and that girls are a little more open about their lack of abilities, knowledge, and about expressing worries about the transfer. However, the girls reported to a larger extent than boys that they take responsibility for talking on the phone with HCPs. In a study by Eaton et al., the opposite was shown since they concluded that girls were more ready to transfer and had less parent involvement than boys [33]. We speculate that this might be due to culture and/or contextual differences, which makes measuring readiness among adolescents in specific contexts and cultures extra important, to enable support to be tailored according to specific needs. In a Swedish study by Burström et al. [26], the aims were to investigate levels of readiness for transition in adolescents with congenital heart disease and to compare adolescents’ levels with their parents’ assessments. Similarly to our study, they demonstrated that adolescents scored higher on overall readiness than their parents. However, they did not compare girls’ and boys’ readiness but investigated differences in perceptions between mothers and fathers. The results show that parents, regardless of gender, perceive adolescents’ responsibility equally. However, perceptions of parental involvement differed between parental genders, meaning that mothers to a greater extent than fathers, perceived themselves as involved [26]. We would argue that this again indicates that differences in perceptions of transition readiness depend on contexts, and that it is important to study the specific population in order to offer tailored support.

Another interesting question about concepts that might have influenced our results, is how the participants interpreted responsibility. If a parent asks an adolescent to call and renew a prescription and the adolescent does so, the adolescent will probably feel that they have taken responsibility, but in fact, the parent was the one responsible for checking that the prescription needed to be renewed and arranging for it to be done. The meaning and interpretation of responsibility for different age groups and parents would be interesting for further studies, to get deeper knowledge about how to communicate about and support independent responsibility for adolescents.

As a final remark, we would argue that to meet adolescents’ and parents’ needs for transition during adolescence, HCPs in both child and adult healthcare must have adequate training. The HCPs need to have good knowledge of adolescents’ normal development and be able to use it in relation to the difficulties that can arise if they also have a chronic illness. It is also important that HCPs are willing to bring up and talk about sensitive topics, for example, sex, alcohol, and relationships. Studies show that structured transition programs can increase both adolescents’ and parents’ confidence regarding the transfer to adult care [25]. This means that introducing a person-centred transition program does not only mean educating patients and parents, but also ensuring that HCPs have adequate knowledge to enable transition in an optimal and positive way.

### Methodological considerations

This study is based on an anonymous survey sent out to adolescents with JIA, with encouragement for their parents to respond to the parent version. However, we do not have information on whether the adolescent lived with a single parent or both parents. The anonymous feature of the survey makes it impossible to link an adolescent to his or her parent(s). Some adolescents may therefore have answered without any parent answering the parent questionnaire and vice versa. Consequently, we do not know how the parents of a specific adolescent responded, and we cannot determine if there is agreement between adolescents and their parents on an individual level. However, our findings indicate that the responses at the group level are consistent. There is also a possibility that both parents chose to participate and answered one questionnaire each. This makes it impossible to calculate a correct response rate for parents as well as compare the results from the adolescent’s and parent’s perspectives in the same family. On the other hand, the advantage of anonymity is that respondents hopefully felt confident to give truthful answers.

When asking the parents about age and gender, some of the parents reported their age and some reported the adolescent’s age. The reports of age and gender are therefore not reliable for parents and not used in the analysis. Furthermore, we did not ask about the age of the adolescent in the parent questionnaire. This means we cannot ensure that the parents’ results correspond to the same age distribution among adolescents as the adolescents’ results.

Another limitation is that background information other than gender and age is not present. This means that we do not know what kind of JIA the patient had. It might have been interesting to be able to see if patients with a milder disease are more prepared for transferring to adult care than those who have a more severe disease or contrariwise. It would have been valuable to ascertain the duration of the adolescents' diagnoses, as this factor might influence their level of responsibility for managing their disease and their readiness for transitioning to adult healthcare. Additionally, investigating the impact of various family structures on the adolescents' ability to assume responsibility for their disease would have been insightful. For instance, the presence of multiple siblings within a family could potentially affect this ability. However, such analyses were not possible in this study due to the lack of relevant background information. As described in the method section, the decision was made to exclude the questions about responsibility for health in the questionnaire. In the original, Gilleland and colleagues use healthcare, and in Burström’s translated Swedish questionnaire, the word health is used [26]. Since we did not conduct cognitive interviews about what adolescents with JIA refer to and include when thinking about health, we assessed it was more scientifically rigorous to exclude the five questions about health. In future studies, it would be interesting to explore this further in cognitive interviews with adolescents, and thereby find out if it is most suitable to ask about health versus healthcare.

In the combined measure of independent responsibility, no distinction was made between the parent being involved sometimes or not at all. The reason for this is that it seems reasonable that parents are sometimes involved in visits to the clinic or talking to the staff, even if the adolescent takes most of the responsibility. The factor analysis and the two latent factors are based on summaries of the scores (1–4) of the items in each factor. Summarizing ordinal data is not optimal but is used here to give a rough picture of the two different factors in this rather small amount of material. In a future larger study, Rasch analysis could be used to develop a validated measurement scale for independent responsibility.

Lastly, we would like to point out that it is difficult to state the results in the sudy are representative of the population concerned since we have no data on JIA subtypes, for example. Moreover, it is probable that the participants who responded are those who felt most concerned or more comfortable with the subject. However, the results in this study based on the rather large sample of 106 adolescents shows strong indications of levels of readiness in this population. In future research stratified sampling could be used to ensure that each subgroup is adequately represented.

### Clinical implications

The results from this study can be used as a foundation for structuring a transition program for adolescents with JIA, including tailoring transition care and creating opportunities for HCPs to focus on the parts of the transition that are perceived as challenging by the adolescents.

Based on the findings of the present study, it is evident that HCPs working in paediatric care have to provide the adolescents and their parents with information and knowledge so that they can feel safe when transferring to adult care. To guarantee an optimal transition from paediatric to adult care, it is imperative to comprehend the special needs of each adolescent, and acknowledge cultural and contextual differences. The RTQ can be used as a screening tool to discover individual needs.

## Conclusion

The results of this study show that adolescents need more support to feel ready to take responsibility for specific healthcare-related behaviours and transfer to adult care. The results indicated that the healthcare behaviours most difficult to take responsibility for included adolescents having to make direct contact with healthcare. The parents also perceived that this was the area that was most difficult for the adolescents to take responsibility for. It is important to pay attention to possible gender differences as well as contextual and cultural differences. The RTQ may be a relevant tool to screen for individual needs during the transition process. With the results from this study, we can customize, and thus optimize, transitional care in Sweden for adolescents with JIA.

## Data Availability

The datasets supporting the conclusions of this article are included within the article.

## Abbreviations

CI: Confidence interval
FS: Factor score
HCP: Health Care Professionals
JIA: Juvenile Idiopathic Arthritis
RTQ: Readiness for Transition Questionnaire
